# Predictors of re-attendance at biennial screening mammography following a false positive referral: A study among women in the south of the Netherlands

**DOI:** 10.1016/j.breast.2024.103702

**Published:** 2024-02-27

**Authors:** Adri C. Voogd, Zsófi Molnar, Joost Nederend, Robert-Jan Schipper, Luc J.A. Strobbe, Lucien E.M. Duijm

**Affiliations:** a Department of Epidemiology, Maastricht University, PO Box 616, 6200 MD, Maastricht, the Netherlands; b Department of Radiology, Catharina Hospital, Michelangelolaan 2, 5623 EJ, Eindhoven, the Netherlands; c Department of Surgery, Catharina Hospital, Michelangelolaan 2, 5623 EJ, Eindhoven, the Netherlands; d Department of Surgery, Canisius Wilhelmina Hospital, Weg Door Jonkerbos 100, 6532 SZ, Nijmegen, the Netherlands; e Department of Radiology, Canisius Wilhelmina Hospital, Weg Door Jonkerbos 100, 6532 SZ, Nijmegen, the Netherlands

**Keywords:** Breast carcinoma, Mammography, Screening, Population-based, False-positive test, Follow-up, Attendance

## Abstract

**Aim:**

A false positive (FP) referral after screening mammography may influence a woman's likelihood to re-attend the screening program. The impact of having a FP result in the first or subsequent screening round on re-attendance after a FP result was investigated. In addition, we aimed to study differences in re-attendance rates between women who underwent non-invasive and invasive additional examinations as part of the diagnostic work-up following a FP referral.

**Methods:**

A consecutive series of 13,597 women with a FP referral following biennial screening mammography in the south of the Netherlands between 2009 and 2019 was included.

**Results:**

The screening re-attendance rate was 81.2% after a FP referral, and 91.3% when also including women who had clinical mammographic follow-up. Women who received a FP referral in the first screening round were less likely to re-attend the screening programme in the following three years, compared to those with a FP test in any subsequent round (odds ratio (OR): 0.59, 95%-confidence interval (CI): 0.51–0.69). Women with a FP referral who underwent invasive examinations after referral were less likely to re-attend the screening programme than those who only received additional imaging (OR, 0.48; 95% CI 0.36–0.64).

**Conclusion:**

Women with a FP referral are less likely to re-attend the screening programme if this referral occurs at their first screening round or when they undergo invasive diagnostic workup. Hospitals and screening organizations should prioritize informing women about the importance of re-attending the programme following a FP referral.

## Highlights

• Of all screened women more than 70% experience a false positive (FP) referral.
• The screening re-attendance rate after a FP referral was 81.2%.
• An additional 10.1% underwent clinical mammographic follow-up instead.
• Women with a FP referral in the first screening round were less likely to re-attend.
• Women should be encouraged to attend regular screening following a FP referral.

## 1 Introduction

The prognosis of breast cancer is strongly dependent on the extent of the disease at diagnosis [1]. Early detection of disease by organized screening of the general population with mammography has a pivotal role in decreasing breast cancer-related death [2].

In general, when a tumour or high-risk lesion is suspected, the woman will be referred to a multidisciplinary breast cancer clinic for additional examinations. In the Netherlands, 71% of the referred women turned out to have received a false positive (FP) referral in 2019, thus yielding a positive predictive value of screening mammography of 29% [3]. Women with a FP breast cancer screening outcome are more likely to experience anxiety and have a lower quality of life, often lasting for more than one year [[4], [5], [6]]. Another important consequence of a FP referral is that these women tend to avoid breast cancer screening programmes after this negative experience with breast cancer screening. Theoretically, this behaviour might lead to a delay in breast cancer diagnosis and eventually an adverse prognosis. Studies in the Netherlands [7], the UK [5], and Canada [8] confirm that attendance rates in future screening rounds is lower after a FP screening outcome, as compared to after a negative screening result. Several studies also found that women with a FP mammogram have a significantly higher risk of developing breast cancer compared to those not having a prior FP test, suggesting that it might be beneficial to actively encourage women with FP tests to continue to attend regular screening [9,10].

This study aimed to investigate possible differences in re-attendance rates between women who experienced their FP mammogram in the first screening round or in any of their subsequent screening rounds. In addition, we aimed to study differences in re-attendance rates between women who underwent non-invasive and invasive additional examinations as part of the diagnostic work-up following a FP referral, and between women who showed consistent and less consistent screening adherence behaviour before experiencing their FP screening mammogram.

## 2 Patients and methods

### 2.1 Study design and population

The Dutch screening programme offers free biennial screening mammography to women aged 50–75 years. We included a consecutive series of women who were screened in a Southern screening region of the Netherlands from January 1, 2009 until January 1, 2020. Prior to attendance, women gave informed consent that their data could be used for quality assurance of the screening programme and for scientific purposes. Women could refrain from this consent using an opt-out construction. Only three referred women did so and they were excluded from analysis. Ethical approval was not required for the current study, according to the Dutch Central Committee on Research involving Human Subjects (CCMO).

Details of our screening programme have been reported previously [7]. In summary, the screening mammograms were obtained at four specialized screening units (three mobile units and one fixed unit) by specialized radiologic technicians. Screen-film mammography was gradually replaced by digital screening mammography in 2009 and 2010. All screening examinations were double read by certified screening radiologists. Non-blinded double reading (the second reader is not blinded to the first reader's opinion) was replaced by blinded double reading in 2015.

### 2.2 Diagnostic work-up and follow-up of recalled women

A woman was referred to a hospital for further analysis if suspicious findings were detected at screening mammography. Diagnostic workup took place in thirty hospitals by multidisciplinary breast cancer teams. The workup of 97.5% of the women was done in six regional hospitals in the south of the Netherlands and the remaining 2.5% in hospitals elsewhere in the Netherlands. After referral, additional breast imaging was performed at the hospital where the woman was referred to and the findings were classified according to the Breast Imaging Reporting and Data System (BI-RADS) [11]. This classification was based on subjective assessment by the clinical radiologists. BI-RADS 4 (suspicious) and BI-RADS 5 (malignant) lesions were routinely biopsied. BI-RADS 3 lesions (probably benign) could undergo either biopsy or radiological follow-up, dependent on shared decision between the clinician and the patient. A FP referral was defined as no diagnosis of breast cancer at workup.

### 2.3 Data collection

The data used in this study are drawn from hospital records, which were collected by the coordinating radiologist (LEMD) and radiology residents, who yearly visited the hospitals who performed the evaluation of referred women. Hospital files contained essential information such as type and outcome of their additional examinations, use of hormonal replacement therapy, and family history of breast cancer. The mammographic breast density, and breast imaging outcome were obtained from the imaging reports and outcome of biopsies and surgery were obtained from the pathology and surgery reports. Information about earlier breast surgery was retrieved from questionnaires filled out by the women during their screening visit. Information on the number of previously missed screening rounds was derived from the screening platform (IBOB). For a follow-up period of three years after the FP event, the coordinating radiologist retrieved information about the re-attendance status of all women, or if they underwent mammography in a clinical setting, either as part of the routine follow-up in the hospital or on the request of the women's general practitioner.

### 2.4 Statistical analyses

In the current study, screening re-attendance was defined as attending the national breast cancer screening programme within three years following a FP referral. Clinical follow-up was defined as not re-attending the screening programme in this three-year period, but instead undergoing mammography and/or more invasive examinations in the hospitals. This could be initiated either after a referral by a general practitioner or as part of a clinical follow-up process*.* Throughout this paper it will be specified whether clinical follow-up is included or not when analysing re-attendance rates.

Women who had died, turned 76 years after the latest screening, had severe comorbidity or diagnosed with breast cancer, were considered as “non-eligible for re-attendance”.

Separate analyses were performed for the women who re-attended breast cancer screening or received clinical follow-up to identify predictors for clinical follow-up.

In line with our research aims, the women with a FP referral were subdivided according to the screening round in which the FP referral took place, the invasiveness of the additional diagnostic procedures that was performed in the hospital to which they were referred and their prior adherence to the screening programme (i.e., having skipped one or more screening rounds). For the subdivision according to screening round, a distinction was made between women who received their FP result in their first round versus any subsequent round. For the subdivision according to the invasiveness of the diagnostic procedures, the following categories were defined: non-invasive diagnostic workup (only imaging), percutaneous biopsy (fine needle aspiration biopsy, core needle biopsy, or stereotactic biopsy) and surgical biopsy. Finally, for the subdivision according to previous screening attendance behaviour a distinction was made between consistent attendees who did not miss any screening round, moderately inconsistent attendees, who had missed only one previous screening round, and inconsistent attendees, who had not attended two or more previous screening rounds. Assessing consistency of screening behaviour was only possible in women who underwent at least two screening rounds during the study period.

To study inter-hospital variation in screening re-attendance, seven subgroups were created, existing of the six hospitals in the Southern screening region of the Netherlands and a seventh group, defined as ‘other’, consisting of the 24 hospitals elsewhere in the Netherlands.

After the descriptive presentation of cohort characteristics, associations between different screening features and re-attendance rates were examined using Chi-square tests. Multivariable binary logistic regression analyses of re-attendance were performed, including the covariates as shown in Table 1. A backward, stepwise procedure was used in which all terms, which were not related significantly (p-value <0.05) to the outcome, were removed. Women with missing data were excluded from the analysis.

**Table 1:** Descriptive characteristics and re-attendance status of women with a false positive (FP) referral.

	All women	Re-attendance	P-value	Re-attendance, including clinical follow-up	P-value
	No. (%)	No. (%)		No. (%)	
**Screening round of FP referral**			<0.001		<0.001
First round	4019 (31.2)	3131 (77.9)		3581 (89.1)	
Subsequent round	8859 (68.8)	7331 (82.8)		8184 (92.4)	
**Screening adherence**^a^			<0.001		<0.001
Consistent	8297 (93.7)	6974 (84.1)		7744 (93.3)	
Moderately inconsistent	378 (4.3)	265 (70.1)		312 (82.5)	
Inconsistent	184 (2.1)	92 (50.0)		128 (69.6)	
**Invasiveness of diagnostic work-up**			<0.001		0.353
Non-invasive	8991 (69.8)	7520 (83.6)		8199 (91.2)	
Small invasive	3654 (28.4)	2779 (76.1)		3348 (91.6)	
surgical biopsy	233 (1.8)	163 (70.0)		218 (93.6)	
**Breast density**			<0.001		0.142
0–25%	3683 (28.6)	3060 (83.1)		3380 (91.8)	
25–50%	6178 (47.9)	5063 (82.0)		5640 (91.4)	
50–75%	2702 (21.0)	2098 (77.6)		2446 (90.5)	
75–100%	319 (2.5)	241 (75.5)		299 (93.7)	
**Age group (years)**			0.78		0.004
49–53	5011 (38.9)	4017 (80.2)		4542 (90.6)	
54–58	2379 (18.5)	1957 (82.3)		2216 (93.1)	
59–63	2016 (15.7)	1642 (81.4)		1852 (91.9)	
64–68	1946 (15.1)	1610 (82.7)		1774 (91.2)	
69–74	1526 (11.8)	1236 (89.0)		1381 (90.5)	
**Prior breast surgery**			<0.001		0.893
Yes	1143 (8.9)	1055 (75.3)		1043 (91.3)	
No	11735 (91.1)	9407 (81.8)		10722 (91.4)	
**Hormone replacement therapy**			0.54		0.551
Yes	479 (3.7)	373 (77.9)		434 (90.6)	
No	12399 (96.3)	10089 (81.4)		11331 (91.4)	
**Family history**			0.007		0.252
Yes	1344 (10.4)	1055 (78.5)		1239 (92.2)	
No	11534 (89.6)	9407 (81.6)		10526 (91.3)	
**Hospital**			<0.001		0.005
1	1225 (9.5)	854 (69.7)		1134 (92.6)	
2	580 (4.5)	483 (83.3)		536 (92.4)	
3	3221 (25.0)	2665 (82.7)		2899 (90.0)	
4	3239 (25.2)	2682 (82.8)		2946 (91.0)	
5	3047 (23.7)	2535 (82.2)		2817 (92.5)	
6	1239 (9.6)	970 (78.3)		1141 (92.1)	
Other	327 (2.5)	273 (83.5)		292 (89.3)	
**Screening period**			<0.001		<0.001
2009–2011	2807 (21.8)	2191 (78.1)		2608 (92.9)	
2012–2014	4961 (38.5)	4145 (83.6)		4595 (92.6)	
2015–2017	3670 (28.5)	3033 (82.6)		3347 (91.2)	
2018–2019	1440 (11.2)	1093 (75.9)		1215 (84.4)	

a Only women who had at least two mammography results.

All analyses were performed with SPSS 26 statistical programme setting the significance level of 0.05 [12].

## 3 Results

### 3.1 Baseline characteristics

**Fig. 1:**
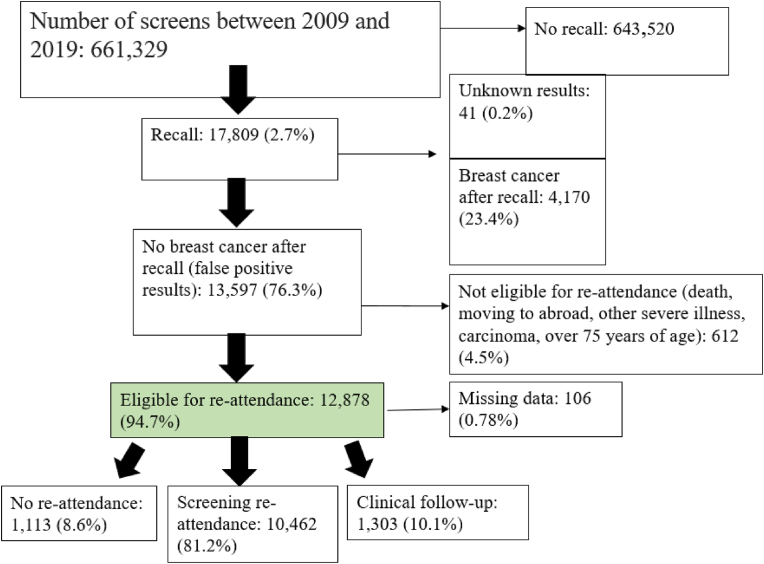
Mammography screening re-attendance in the source population between January 2009 and 2019.

From 2009 until 2020,661,329 screening examinations were performed, and 17,809 women (2.7%) were referred to a hospital for further examination because of a suspicious finding. Of these 17,809 referred women, 13,597 (76.3%) experienced a FP referral, of whom 12,878 were identified as eligible to re-attend the screening programme and 106 were excluded because of missing data. (Fig. 1). The overall re-attendance rate among women with a FP referral was 81.2% (not including clinical follow-up) and 91.3% when including a subsequent mammography ordered by a general practitioner or when under hospital surveillance (Fig. 1). Of the women with a FP screening mammogram 4019 (31.2%) received this outcome at their first screening round and 8859 women (68.8%) at one of the subsequent screening rounds. The mean age for the women at the time of their FP screening mammogram was 58.1 years (SD: 7.2 years).

Considering the diagnostic work-up after mammography screening, 69.8% of women with a FP result underwent non-invasive imaging only, 28.4% underwent percutaneous biopsy and 1.8% surgical biopsy (with or without previous percutaneous biopsy). Of the 8859 women who had attended at least two screening rounds, 93.7 % showed consistent attendance and participated in every screening round for which they were invited, 4.3% showed moderately inconsistent attendance (having missed one round) and 2.1 % had missed two or more rounds. (Table 1).

### 3.2 Re-attendance: first round versus subsequent screening rounds

Of the women with a FP referral in the first screening round, 77.9% re-attended the screening programme in the following three years, compared to 82.8% of the women experiencing a FP referral in one of the subsequent screening rounds (P < 0.001) (Table 1). When including clinical follow-up, the re-attendance rates were 89.1% and 92.4% respectively (P < 0.001). Also, after adjustment for potential confounders in the multivariable analysis, women with a FP referral in the first screening round were less likely to re-attend the screening programme, with an odds ratio (OR) of 0.59 (95% CI 0.51–0.69) (Table 2). The OR was 0.47 (95% CI 0.38–0.59) when clinical follow-up was also included.

**Table 2:** Multivariable analyses of the role of first versus subsequent screening round and invasiveness of the diagnostic examination after false positive mammography result on screening re-attendance, including covariates (n = 12,878).

	Re-attendance		Re-attendance, including clinical follow-up	
	OR (95% CI)	*P*-value	OR (95% CI)	*P*-value
**Screening round of FP referral** Subsequent screening round	1 (Reference)		1 (Reference)	
First screening round	0.59 (0.50–0.69)	<0.001	0.47 (0.38–0.59)	<0.001
**Invasiveness of diagnostic work-up**				
Non-invasive	1 (Reference)		1 (Reference)	
Small-invasive	0.64 (0.58–0.71)	<0.001	1.07 (0.93–1.23)	0.336
Excision biopsy	0.48 (0.36–0.64)	<0.001	1.49 (0.88–2.54)	0.139
**Breast density**				
0–25%	1 (Reference)			
25–50%	0.91 (0.82–1.02)	0.107		
50–75%	0.75 (0.66–0.75)	<0.001		
75–100%	0.69 (0.52–0.91)	0.008		
**Prior breast surgery (yes vs. no)**	0.69 (0.6–0.8)	<0.001		
**Family history (yes vs. no)**	0.82 (0.71–0.95)	0.006		
**Hospital**				
1	1 (Reference)		1 (Reference)	
2	2.03 (1.58–2.62)	<0.001	1.09 (0.75–1.59)	0.646
3	2.08 (1.79–2.43)	<0.001	0.75 (0.59–0.95)	0.019
4	2.02 (1.74–2.36)	<0.001	0.81 (0.63–1.04)	0.091
5	2.14 (1.83–2.50)	<0.001	0.99 (0.77–1.28)	0.938
6	1.54 (1.28–1.86)	<0.001	0.98 (0.73–1.32)	0.879
Other	2.18 (1.58–2.99)	<0.001	0.71 (0.47–1.07)	0.103
**Age group (years)**				
49–53	1 (Reference)		1 (Reference)	
54–58	0.76 (0.64–0.9)	0.001	0.8 (0.62–1.02)	0.074
59–63	0.68 (0.57–0.82)	<0.001	0.62 (0.48–0.81)	<0.001
64–68	0.74 (0.61–0.89)	0.002	0.57 (0.41–0.74)	<0.001
69–74	0.67 (0.55–0.82)	<0.001	0.53 (0.40–0.69)	<0.001

OR Odds Ratio, CI confidence interval.

### 3.3 Re-attendance: non-invasive versus invasive diagnostic procedures

Screening re-attendance rates decreased with increasing invasiveness of the diagnostic procedures following a FP referral. Of the women undergoing non-invasive diagnostic work-up (i.e., imaging alone) 83.6% re-attended the screening programme, compared to 76.1% of the women undergoing percutaneous biopsy and 70% of the women undergoing surgical biopsy (P < 0.001) (Table 1). In a multivariable model odds ratios were 0.64 (95% CI 0.58-0-71) and 0.48 (95% CI 0.36–0.64) respectively, compared to the women who underwent imaging alone. When clinical follow-up was included, no statistically significant differences were observed in re-attendance rates between patients with a non-invasive diagnostic work, percutaneous biopsy or excision biopsy, neither in the univariate analysis (Table 1), nor in the multivariable model (Table 2).

### 3.4 Re-attendance: consistent versus inconsistent attenders

Consistency of screening attendance was only measurable in women who participated in at least two screening rounds. Differences in re-attendance rates between consistent, moderately inconsistent and inconsistent attenders were statistically significant when considering the absolute percentages: 84.1%, 74.1% and 70%, respectively, of women with FP referral re-attended the screening programme (P < 0.001) (Table 1). After adjustment for relevant co-variates in the multivariable analysis (Table 3), the OR was 0.45 (95% CI 0.36–0.57) for the moderately inconsistent and 0.18 [95% CI 0.14–0.25) for these least consistent attenders. When including clinical follow-up, crude re-attendance percentages of 93.3%, 82.5% and 69.6% were found (P < 0.001) (Table 1), while after adjustment for co-variates, the odds ratios were 0.33 (95% CI 0.25–0.43) and 0.16 (95% CI 0.11–0.23), respectively (Table 3).

**Table 3:** Multivariable analyses of the role of screening adherence after false positive mammography result on screening re-attendance, including covariates (n = 8859).

	Re-attendance		Re-attendance, including clinical follow-up	
	OR (95% CI)	*P*-value	OR (95% CI)	*P*-value
**Screening adherence** Consistent attendees	1 (Reference)		1 (Reference)	
Moderately inconsistent attendees	0.45 (0.36–0.57)	<0.001	0.33 (0.25–0.43)	<0.001
Inconsistent attendees	0.18 (0.14–0.25)	<0.001	0.16 (0.11–0.23)	<0.001
**Breast density**				
0–25%	1 (Reference)			
25–50%	0.96 (0.84–1.09)	0.498		
50–75%	0.86 (0.73–1.01)	0.068		
75–100%	0.61 (0.43–0.87)	0.007		
**Prior breast surgery (yes vs. no)**	0.82 (0.68–0.99)	0.035	1.3 (0.97–1.74)	0.094
**Family history (yes v. no)**	0.84 (0.71–1.01)	0.058		
**Hospital**				
1	1 (Reference)			
2	1.73 (1.28–2.34)	<0.001		
3	2.16 (1.79–2.62)	<0.001		
4	2.09 (1.73–2.54)	<0.001		
5	2.21 (1.82–2.68)	<0.001		
6	1.62 (1.29–2.04)	<0.001		
Other	2.87 (1.85–4.44)	<0.001		
**Invasiveness of diagnostic work-up**				
Non-invasive	1 (Reference)		1 (Reference)	
Small-invasive	0.63 (0.56–0.72)	<0.001	1.09 (0.91–1.31)	0.351
Excision biopsy	0.54 (0.37–0.79)	<0.001	2.58 (1.05–6.35)	0.039
**Age group (years)**				
49–53			1 (Reference)	
54–58			1.30 (0.98–1.73)	0.072
59–63			1.01 (0.76–1.34)	0.954
64–68			0.91 (0.69–1.20)	0.506
69–74			0.81 (0.61–1.08)	0.160

OR Odds Ratio, CI confidence interval.

### 3.5 Predictors of clinical follow-up

A multivariable analysis was performed to identify predictors for women of undergoing clinical follow-up following a FP referral (Table 4). Women with a FP referral in their first screening round were more likely to receive clinical follow up, as compared to women who experienced a FP referral in a subsequent round (OR 1.35; 95% CI 1.09–1.65). Women whose diagnostic work-up consisted of any kind of biopsy were also significantly more likely to undergo clinical follow-up, as compared to the women undergoing non-invasive diagnostic procedures; the OR was 2.15 (95% CI 1.90–2.44) for women receiving percutaneous biopsy and 3.82 (95% CI 2.75–5.31) for those undergoing surgical biopsy. Other factors that were positively associated with the likelihood to undergo clinical follow-up were breast density, having undergone breast surgery in the past and having a positive family history for breast cancer. Compared to reference hospital 1, women with a FP referral to the other 5 hospitals and the ‘other’ category, representing the remaining 24 hospitals, were significantly less likely to undergo clinical follow-up.

**Table 4:** Predictors for clinical follow-up (either after referral by a GP or as part of clinical follow-up in the hospital) in women with false positive (FP) findings at screening mammography re-attending the screening programme or undergoing clinical follow-up (n = 11,765).

	OR (95% CI)	P-value
Screening round of FP referral		
Subsequent screening round	1 (Reference)	
First screening round	1.35 (1.09–1.65)	0.005
**Invasiveness of diagnostic work-up**		
Non-invasive	1 (Reference)	
Small invasive	2.15 (1.91–2.44)	<0.001
Excision biopsy	3.82 (2.75–5.31)	<0.001
**Breast density**		
0–25%	1 (Reference)	
25–50%	1.30 (1.11–1.51)	<0.001
50–75%	1.66 (1.4–1.98)	<0.001
75–100%	2.15 (1.55–2.97)	<0.001
**Hospital**		
1	1 (Reference)	
2	0.41 (0.30–0.57)	<0.001
3	0.26 (0.21–0.31)	<0.001
4	0.30 (0.25–0.36)	<0.001
5	0.32 (0.26–0.38)	<0.001
6	0.57 (0.46–0.71)	<0.001
Other	0.22 (0.13–0.36)	<0.001
**Family history (yes vs. no)**	1.52 (1.27–1.81)	<0.001
**Prior breast surgery (yes vs. no)**	1.78 (1.49–2.13)	<0.001
**Age group (years)**		
49–53	1 (Reference)	
54–58	1.34 (1.07–1.68)	0.010
59–63	1.32 (1.04–1.67)	0.024
64–68	1.07 (0.83–1.38)	0.588
69–74	1.18 (0.91–1.53)	0.277
	**OR (95% CI)**	**P-value**
**Screening period**		
2009–2011	1 (Reference)	
2012–2014	0.55 (0.47–0.64)	<0.001
2015–2017	0.50 (0.42–0.59)	<0.001
2018–2019	0.55 (0.44–0.69)	<0.001

OR Odds Ratio, CI confidence interval.

## 4 Discussion

In the current study, we focused on the effect of a FP referral at screening mammography on the likelihood to re-attend the screening programme. We found that a vast majority of women with a FP referral received a mammogram within three years after their referral, either by participating in the screening programme or by undergoing clinical follow-up in a hospital. We did separate analyses for the women who received a FP referral in their first screening round versus those with FP results in any of the subsequent rounds and found that re-attendance was significantly lower for the first group, also after considering potential confounders. Moreover, women with a FP result who underwent invasive diagnostic procedures were less likely to re-attend the screening programme than those who only received non-invasive examinations to rule out breast cancer. However, many of these women underwent clinical follow-up instead of screening mammography. We observed a significantly lower willingness to re-attend the screening programme among women who had missed one or more screening rounds prior to their FP referral.

In our study, information on re-attendance was only available for women with a FP referral and not for those with a normal mammogram, not requiring further diagnostics. Recent figures show that the re-attendance rate in women with a negative screening mammogram was 93.8% in the Netherlands in 2020, which was higher than the 91.3% re-attending the programme or undergoing clinical follow-up in our study, following a FP referral [3]. The lower re-attendance rate in the screening programme among women who experienced a FP referral in the first screening round may be due to their lack of experience with mammography, in combination with a negative first impression of the screening process. A FP referral has been described to cause long-term psychological harm [[4], [5], [6]], and this harm and its impact on re-attendance may be larger in women participating in the screening programme for the first time. Our finding of a lower overall screening re-attendance rate among women with a FP screening mammogram at their first screening round is in accordance with the findings of studies from Spain and the UK [13,14]. However, in another study from the UK by Maxwell et al. women who experienced a FP referral in their first screening round were found to be significantly more likely to re-attend than those with a normal screen (87.7% vs. 86.0%) and no significant difference in re-attendance rate was observed in any of the subsequent screening rounds between women who had a FP screening examination and those with a normal screen [15]. Also in a study from Denmark, no significant difference was observed in participation in the subsequent screening rounds between women with a FP screening test and women with a negative test [16]. Possible explanations for these divergent results could be differences between countries or regions in the level of centralization of breast cancer care, availability of widely accepted guidelines when to opt for clinical follow-up or when to send a woman back to the screening programme, and whether costs related to clinical follow-up are reimbursed or not.

Women who underwent invasive diagnostic procedures after FP referral appeared to be more likely to undergo follow-up in the hospital. This is in accordance with the results of the previously mentioned study by Maxwell et al. [15], in which women who underwent needle sampling or open biopsy following a FP referral in any of the incident screening rounds were 12% and 60% less likely to re-attend, respectively, than women whose screening examinations were normal. We also showed that the decision not to refer women back to the screening programme but offer them mammography in the hospital was related to factors such as a family history of breast cancer and the density of their breast tissue, which are established risk factors of breast cancer [17]. These women may have been offered alternative imaging modalities, such as MRI and ultrasonography [18,19]. Other factors which might have influenced the choice for clinical follow-up, but on which we have no information available in our study, are an elevated breast cancer risk based on the biopsy findings and personal preferences of the referred women and the multidisciplinary breast cancer team performing the diagnostic work-up. Together, these factors probably also explain part of the observed variation between hospitals, which was generally small, except for one hospital.

We found that moderately inconsistent and inconsistent screening attendees were significantly less likely to re-attend at screening mammography. In a recent study Duffy et al. demonstrated the importance of re-attending screening mammography at regular intervals. They followed 549,091 Swedish women during two screening rounds and showed that serial participants, which are the women who participated in the last two screening mammography rounds prior to breast cancer diagnosis, had a 50% lower risk of death from breast cancer within 10 years of diagnosis (relative risk, 0.50; 95% CI 0.46, 0.55; P < 0.001) than serial non-participants, which are the women who missed both screening rounds. We subscribe their important message for women in the screening age groups, their referring physicians, and public health decision makers, that missing even one screening examination confers a significant increase in the risk of dying from breast cancer [20].

Our study has several strengths and limitations. To our knowledge, it is the first analysis of previous attendance behaviour and numerous factors influencing re-attendance after a FP referral, in a large study population with virtually complete information on screening re-attendance and diagnostic procedures following referral. A limitation is that we did not collect information on women's personal experiences with and feelings towards the screening programme in general and the course of events following a FP referral. Establishing further potential factors on re-attendance, such as breast cancer-related anxiety after receiving a FP result or painful experience at additional workup were beyond the scope of this study, but several studies have already shown that these issues can have a negative impact on women's willingness to re-participate [21]. Since our study took place in a screening mammography region of the Netherlands, generalizability of our findings is challenging as the setup of screening programmes and referral procedures varies among countries. Finally, it should be noted that our study involves a large number of women, which means that small, possibly irrelevant differences will also become statistically significant. Clinical relevance of the absolute differences can, to some extent, be judged by the re-attendance rates presented in Table 1.

## 5 Conclusion

We identified several factors that influenced the probability of screening re-attendance after a FP referral. Women with a FP recall are less likely to re-attend the screening program if this recall occurs in their first screening round or when they had skipped one or more screening rounds in the past. Hospitals and screening organizations should prioritize informing women about the importance of re-attending the programme following a FP referral.

## Ethics approval

Ethical approval was not required for the current study, according to the Dutch Central Committee on Research involving Human Subjects (CCMO).

## Funding

None.

## Data statement

The datasets used and/or analysed during the current study are available from the corresponding author on reasonable request.

## CRediT authorship contribution statement

**Adri C. Voogd:** Conceptualization, Methodology, Writing – original draft, Writing – review & editing, Supervision. **Zsófi Molnar:** Conceptualization, Formal analysis, Writing – original draft. **Joost Nederend:** Conceptualization, Writing – review & editing. **Robert-Jan Schipper:** Conceptualization, Writing – review & editing. **Luc J.A. Strobbe:** Conceptualization, Writing – review & editing. **Lucien E.M. Duijm:** Conceptualization, Data curation, Methodology, Supervision, Writing – review & editing.

## Declaration of competing interest

None.
